# Emerging Landscapes of Tumor Immunity and Metabolism

**DOI:** 10.3389/fonc.2020.575037

**Published:** 2020-10-07

**Authors:** Fan Wu, Ye Cheng, Liangliang Wu, Wenling Zhang, Wubing Zheng, Qian Wang, Hongyong Cao, Xiongxiong Pan, Weiwei Tang

**Affiliations:** ^1^Department of General Surgery, Nanjing First Hospital, Nanjing Medical University, Nanjing, China; ^2^Department of Gastroenterology, The First Affiliated Hospital of Nanjing Medical University, Nanjing, China; ^3^Research Unit Analytical Pathology, Helmholtz Zentrum München, German Research Center for Environmental Health, Neuherberg, Germany; ^4^Department of Anesthesiology, The First Affiliated Hospital of Nanjing Medical University, Nanjing, China

**Keywords:** cancer, metabolism, immunity, reprogram, drug resistance

## Abstract

The metabolic reprogramming of cancer tissue has higher metabolic activity than surrounding tissues. At the same time, the local infiltration of immunosuppressive cells is also significantly increased, resulting in a significant decrease in tumor immunity. During the progression of cancer cells, immunosuppressive tumor microenvironment is formed around the tumor due to their metabolic reprogramming. In addition, it is the changes in metabolic patterns that make tumor cells resistant to certain drugs, impeding cancer treatment. This article reviews the mechanisms of immune escape caused by metabolic reprogramming, and aims to provide new ideas for clinical tumor immunotherapy combined with metabolic intervention for tumor treatment.

## Introduction

Current research on the immune system’s defense against cancer has led to the rapid development of cancer immunotherapy. Some cancer cells are immunogenic due to their high mutation rate, leading to immune escape (1). Immune escape is considered a marker of cancer progression, highlighting the direct involvement of immune cells (2). Immune cells affect all aspects of cancer progression, including survival, proliferation, angiogenesis, and metastasis (3). The immune escape produced by cancer cells is accomplished by reediting the immune system. Immune editing is a dynamic process that includes elimination, balance, and escape. First, tumor cells interfere with the immune system to eliminate rejection of cancer cells. Then, the immune system cannot completely eliminate the tumor cells to reach a state of equilibrium. Finally, with the emergence of new tumor cell variants, an immunogen was selected. Cells with low resistance or immune resistance reach the final immune escape stage (2). Meanwhile, recent studies have shown that immune cells have unique metabolic characteristics that affect their immune function, and metabolic reprogramming is an important step in activating immune cells (4). It is these metabolic changes that further influence the immune system during cancer development, which promote the progression of cancer.

## Reprogramming of Metabolism for Cancer Progression

Cancer cells are well-known for their faster growth and proliferation than normal cells, and in order to meet such demands, they must reprogram cell’s metabolism (5). Glucose metabolism produces ATP mainly through the oxidation of its carbon bonds. The end product can be lactic acid (anaerobic glycolysis pathway) or carbon dioxide (oxidative phosphorylation pathway) after glucose is completely oxidized by mitochondrial respiration. In tumor cells, even in the presence of oxygen and fully functioning mitochondria, glucose uptake rates increase dramatically and lactic acid is produced, known as the Warburg effect (6). Although the amount of ATP produced by glycolysis is small, its production rate is much faster than that of oxidative phosphorylation (7). In addition, the Warburg effect of tumor cells leads to increased production of lactic acid and extracellular pH of tumor microenvironment (TME), which is conducive to the processes of metastasis, angiogenesis, and immunosuppression (8). The Warburg effect is caused by a number of mechanisms. For example, glucose transporters mediate glucose transport, the first step in glycolysis, while PTEN and GLUT1 expression in the tumor cytoplasm is inversely correlated. Increased membrane expression and glucose uptake GLUT1, enhancing the Warburg effect (9). In addition, in renal cancer cells lacking von Hippel-Lindau tumor suppressor (VHL), HIF-1 also inhibits metabolism in the tricarboxylic acid (TCA) cycle by directly activating PDK1 (10).

In order to meet the rapid proliferation of cancer cells, the demand for amino acids increased significantly. Increased glutamine metabolism is a common metabolic change. Glutamine catabolism is catalyzed by glutaminase (GLS) to produce glutamic acid. Oncogenic transcription factor cMyc activates GLS expression and metabolism in tumor cells (11).

In cancer cells, the biosynthesis of fatty acids (FA) is more active, and cancer cells typically achieve higher lipid accumulation in the form of lipid droplets than normal cells. After the citric acid is produced by TCA cycle in mitochondria, the citric acid carrier (CIC) is transferred from the inner membrane of mitochondria to the cytoplasm and then enters *de novo* synthesis. CIC levels are elevated in many cancer cells, and its activity is necessary for tumor proliferation *in vitro* and tumorigenesis *in vivo* (12). In summary, tumor cells have reprogrammed their metabolism to affect normal cell metabolism while gaining much greater proliferation capacity than normal cells.

## Glucose Metabolism and Tumor Immunity

T cells are one of the important cells of tumor immunity, and it is necessary to express specific antigen in tumor. The anti-tumor activity of T cells is greatly influenced by cell metabolism. Therefore, in the process of tumor development, metabolic reprogramming of cells inevitably affects the anti-tumor activity of T cells (13). Normally, anaerobic glycolysis is the key to maintaining T-cell immune function (14). When blood glucose is normal, T cells up-regulate glucose transporter 1 and then promote glucose uptake and anaerobic glycolysis when stimulated by antigens (15). In acute infection, apoptotic T cells and memory T cells independent of anaerobic glycolysis are produced and undergo aerobic glycolysis (16).

As previously mentioned, the Warburg effect of tumor cells significantly increases the content of lactic acid in TME, a pro-inflammatory agent that activates the IL-23/IL-17 pathway, leading to inflammation, angiogenesis, and cell remodeling. Meanwhile, the increase of lactic acid in TME leads to the decrease of pH value, and the expression of arginase I (ARG1) in macrophages increases after the acidification of TME, thus inhibiting the proliferation and activation of T cells (17).

It is well known that programmed death ligand 1 (PD-L1, also known as CD274, and B7-H1) binds to its receptor PD-1 to produce effects. PD-1 is a cell surface protein that is widely present on the surface of T cells, NK cells and dendritic cells (DC) (18). The combination of PD-L1 and PD-1 triggers inhibitory signaling, thereby suppressing the role of T cells (19). Shaojia Wang et al. found that overexpression of PD-L1 in cervical cancer cells increases glucose metabolism and is associated with tumor metastasis. From a mechanistic perspective, PD-L1 directly binds to integrin β4 (ITGB4) and activates the AKT/GSK3β signaling pathway to induce the expression of the transcriptional repressor SNAI1. SNAI1 can affect the epithelial-mesenchymal transition and the expression of genes regulating glucose metabolism by inhibiting the activity of SIRT3 promoter, thereby inhibiting T cell action and promoting tumor immune escape. The high expression of PD-L1 and ITGB4 in human cervical cancer is closely related to T cell function inhibition, tumor lymph node metastasis and poor prognosis (20). Siska Peter J et al. discovered that in patients with B-cell leukemia, the expression of PD-1 and TIM3 will increase, which will cause the activation of T cells, but will also lead to a decrease in T cell reactivity at the same time. Due to the increased expression of PD-1 and TIM3, it can genetically cause a decrease in Akt/mTORC1 signaling or Glut1 expression, resulting in impaired T cell metabolism and inhibiting T cell function (21). Co-stimulation and inhibitory signals jointly regulate the anti-tumor ability of tumor antigen-specific T cells. In the past, we always tried to restore the function of unresponsive T cells by blocking the inhibitory pathway. On the contrast, there have been opinions that provide T cells with extra co-stimulation signals can also enhance its anti-tumor function recently. Polesso Fanny et al. demonstrated a synergistic effect of targeted blockade of PD-L1 and the provision of a co-stimulatory agonist to OX40, which can increase the glucose metabolism of CD8 + T cells and the acquisition of granzyme B by regulatory T cells, which increase The existence and function of tumor antigen-specific CD8^+^T cells (22).

MicroRNA is an important substance regulating T cell immunity (23). Zhang Tengfei et al. examined the effect of miR-143 on the differentiation and function of T cells, and found that in esophageal cancer cell lines, overexpression of miR-143 inhibited the glucose transporter 1 (Glut-1) in T cells, inhibiting the glucose uptake and glycolysis of T cells, thereby regulating the differentiation of T cells and inhibiting the antitumor effect of T cells (24). In addition, Zhao Ende et al. found that ovarian cancer cells restrict the glucose uptake of T cells by maintaining the high expression of miR-101 and miR-26a, which weaken their function and thereby limit their methyltransferase EZH2 expression. Under normal circumstances, EZH2 can inhibit the Notch repressors Numb and Fbxw7 by trimethylating the histone H3 located at Lys27, and then further activate the Notch pathway to stimulate the expression of T cell multi-factors and transmit Bcl-2 signal to promote their survival (25).

Generally speaking, as a part of immune cells, neutrophils are important to provide immune protection to the body. However, in cancer, neutrophils can destroy the function of T cells through reactive oxygen species (ROS), thereby promoting tumor progression. We generally believe that neutrophils rely entirely on glycolysis to produce energy. Research by Rice Christopher M et al. revealed that immature c-Kit neutrophil subsets can participate in oxidative mitochondrial metabolism. In the TME, due to insufficient glucose supply, oxidative neutrophils can oxidize mitochondrial FA to generate NADPH oxidase-dependent ROS, and then inhibit the role of T cells to maintain local immune suppression. Consistent with this, neutrophils in peripheral blood of cancer patients generally show immature status, and the content of mitochondria and the degree of oxidative phosphorylation have increased (26).

A molecule called TIGIT exists on the surface of T cells, which is an immune checkpoint molecule that inhibits T cell responses. He Weiling et al. evaluated the role of TIGIT checkpoints in the occurrence and development of gastric cancer. They found that the proportion of CD8^+^ T cells expressing TIGIT on the surface of gastric cancer patients increased. In addition, gastric cancer tissues and cell lines also expressed CD155, which combined with TIGIT to restrict the glucose uptake of CD8^+^ T cells and then weaken the function of CD8^+^ T cell effector molecules, causing these cells to exhibit functional failure and impairing their activation, proliferation, cytokine production, and metabolism. Once CD155 is silenced, T cell metabolism in gastric cancer tumor cells is more active than before, and IFN-γ production is increased. Similarly, targeting CD155/TIGIT can enhance CD8^+^T cell response and improve the survival rate of experimental animals (27).

In tumor immunotherapy, the biggest obstacle is the immunosuppressive microenvironment induced by regulatory T (Treg) cells. Treg can induce normal cell death and suppress effector T cells by mediating accelerated glucose depletion. Li Lingyun et al. discovered that TLR8 signal transduction can selectively inhibit glucose uptake and glycolysis in Treg, thereby reversing Treg’s inhibitory function, which can be a feasible method to promote tumor immunotherapy (28).

A large number of clinical evidences show that T cell immunotherapy is of great benefit to the prognosis of tumor patients. However, many studies have found that in solid cancers, this type of immunotherapy is often limited due to down-regulation of MHC I antigen presentation. In view of this, the relevant experiments designed by Marijt Cohen et al. showed that the phosphorylation signal transducer STAT1 failed to express itself in tumor cells for the reason that time was an anoxic environment and glucose deficiency, which resulted in a decrease in MHC class speech antigen even in the presence of sufficient stimulating cytokine IFN-γ. In cancer cells under this TME, the activity of PI3K in tumor cells increased, leading to the decreased sensitivity of CD8^+^ T cells to tumor recognition (29). Catalan Elena et al. also found that the deficiency of ERK5 expression and the decrease of MHC I expression in tumor cells made tumor cells more prone to glycolysis, which would help tumor cells to escape the immune monitoring of cytotoxic T cells (CTL). Furthermore, through further research on leukemia EL4 cells and L929-transformed fibroblasts, they also concluded that tumor cells are sensitive to CTL when MHC-1 is at a low expression level in tumor cells. At the same time, the sensitivity to NK cells has been improved. However, when its MHC-1 expression is completely deleted, tumor cells can promote mitochondrial oxidative phosphorylation to increase the efficacy of tumor immunotherapy (30).

Whether fighting infection or defending against cancer, CD8^+^ memory T cells (Tm) are the basis of immunity, whose activity is controlled by metabolic activity. Under normal circumstances, CD8^+^ Tm up-regulates PCK1 in the cytoplasm -a key molecule that regulates glycolysis, tricarboxylic acid cycle, and gluconeogenesis, increases glycolysis and promotes the breakdown of glycogen to glucose 6-phosphate, and then generates a large amount of NADPH through Pentose Phosphate Pathway (PPP) to ensure that glutathione is at a high level in Tm. Ma Ruihua et al. found that the pathway mentioned above were inhibited during the development of tumors, causing the GSH/GSSG ratio in Tm decreased, which result in obstacles to Tm formation and impaired function (31).

**FIGURE 1:**
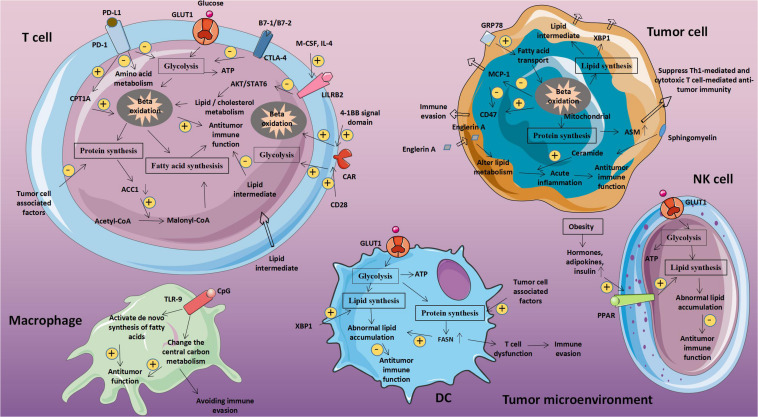
Abnormal glucose metabolism and tumors. Glucose-related metabolic abnormalities in tumor cells can affect the tumor microenvironment. At the same time, tumor cells competitively absorb glucose from the extracellular environment, resulting in decrease in glucose in T cells, thereby inhibiting T cell energy supply, blocking synthesis and affecting T cell function. GLUT1, Glucose transporter 1; MCT-1, Monocarboxylate transporter 1; ASCT2, Amino acid transporter 2; LAT1/LAT2, L-amino acid transporter 1/2; and ROS, Reactive oxygen species.

As shown in Table 1, these molecules are all associated with glucose metabolism and tumor immunity. Glucose-related metabolic abnormalities in tumor cells can affect the TME. At the same time, tumor cells competitively absorb glucose from the extracellular environment, resulting in decrease in glucose in T cells, thereby inhibiting T cell energy supply, blocking synthesis and affecting T cell function (Figure 1).

**TABLE 1:** Glucose metabolism and tumor immunity.

Molecules/drugs	Status	Major effects	Pathway	Tumor types	Type of Immune cells	References
PD-L1	Up	Promote tumor cell glycolysis impede T cell glycolysis and IFN-γ production	AKT/MTOR pathway	Ovarian cancer	T cells	(25)
STAT3 Wnt-β-catenin	Down	Improve long-term survival and anti-tumor activity of T cell	Reduce glycolysis, increase fatty acid oxidation	–	T cells	(88, 89)
HIF	Up	T cell dysfunction	Increase glucose transporter expression, and mTOR activation	–	T cells	(90)
GAPD	Up	T cell dysfunction	Block translation of IFN-γ from mRNA	–	T cells	(91)
Foxp3	Up	TCR-stimulated T cells preferentially differentiate into Tregs	Reduce the expression of Myc, leading to reduced glycolysis and increased oxidative phosphorylation	–	T cells	(92)
CTLA-4	Up	Preventing activation and differentiation of naïve CTLs	CTLA-4 competes for the same ligands as CD28	–	T cells	(93)
Oxamate	–	Arrest the growth of tumor cells	Inhibit LDH production	–	T cells	(94)
AMPK	–	Protect CTLs from apoptosis and promote effector functions	Down-regulate glycolytic gene expression	Colon cancer	T cells	(95)
PD-1 TIM3	Up	T cell dysfunction	Defective Akt/mTORC1 signaling, reduced expression of Glut1 and hexokinase 2, and decreased glucose metabolism	B cell leukemia	T cells	(21)
IRE1α-XBP1	Up	Decrease the ability of T cells to infiltrate tumor tissues and reduce the expression of IFNG mRNA	Inhibit activity of mitochondrial	Ovarian cancer	T cells	(58)
miR-143	Up	Inhibit T cell glucose uptake and glycolysis	Glut-1	Esophageal cancer	T cells	(24)
miR-101 miR-26a	Up	Restrict glucose uptake by T cells	Notch	Ovarian cancer	T cells	(25)
ROS	–	Disrupt the function of T cells	Oxidized mitochondrial fatty acid	–	T cells	(26)
STAT1	Down	CD 8 T cells are not sensitive to tumor recognition	Down-regulation of MHC I antigen presentation	–	CD8^+^ T cells	(29)
PD-L1	Block	Increase the presence and function of tumor antigen-specific CD8 T cells	Glucose metabolism of strong CD8 T cells	–	CD8^+^ T cells	(22)
Lactic acid	Up	Inhibition of T cell proliferation and activation	Increased expression of ARG1 in macrophages	–	T cells	(17)
ERK5 MHC I	Down	Helps tumor cells escape immune surveillance of CTL	Promote glycolysis of tumor cells	–	T cells	(30)
PD-L1 ITGB4	–	Inhibit T cell	AKT/GSK3β	Cervical cancer	T cells	(20)
TIGIT	Up	Limiting glucose uptake by CD8 T cells	CD155 binds TIGIT receptor	Gastric cancer	CD8 ^+^ T cells	(27)
Pck1	Down	Tm formation obstacle	Reduced GSH/GSSG ratio in Tm	–	Tm cells	(31)
TLR8	–	Reverse Treg inhibition	Selective inhibition of glucose uptake and glycolysis in Treg	–	Tregs	(28)

## Fatty Acid Metabolism and Tumor Immunity

### Macrophages and Fatty Acid Metabolism

Macrophages are widely present in all tissues, and have strong plasticity and functional diversity. Macrophages are involved in the initiation and development of a variety of diseases, and therefore have become important intervention targets for diseases. Macrophages mainly depend on glycolytic metabolism capacity, but fatty acid levels in cells can also significantly affect cell function. The protein chaperone glucose-regulated protein 78 (GRP78) can mediate the endoplasmic reticulum (ER) stress pathway and cause an unfolded protein response. Studies on breast cancer cells showed that after the expression of GRP78 on the surface of cancer cells is silenced, fatty acid transport in mitochondria is inhibited, which reduces fatty acid oxidation and increases the concentration of unsaturated FA in the cell. Animal experiments by Cook Katherine L et al. showed that inhibiting GRP78 or reducing linoleic acid content in cells could increase MCP-1 in serum and reduce CD47 expression, thus increasing macrophage infiltration (32).

Macrophages enhance the body’s anti-tumor immunity by phagocytosing and killing tumor cells. Liu Mingen et al. found that cancer cells express CD47 to achieve immune escape, which can be avoided by CpG oligodeoxynucleotides-a Toll-like receptor 9 agonist. CpG oligodeoxynucleotides can change the central carbon metabolism of macrophages. It can enhance the anti-tumor immune activity of macrophages and promote the phagocytosis of CD47 expressing cancer cells by macrophages. Since CpG activates the *de novo* synthesis of FA, it gives macrophages anti-cancer potential (33).

### T Cells and Fatty Acid Metabolism

Studies have shown that the energy supply of T cells mainly depends on anabolic metabolism, such as aerobic glycolysis. But fatty acid metabolism can regulate the immune function activity of T cells, including the balance between effector T cells (Teff) and T cells (34). At the same time, Treg is also involved in the up-regulation of FAO gene expression, and the increase in FAO levels can further promote the generation of Treg cells (35). In addition, FAO plays an important role in the production and maintenance of Tm cells (36).

The development of cancer will lead to changes in lipid metabolism, causing a change in the proportion of metabolic intermediates, which will have a certain impact on the immune system. Wefers Christina et al. collected cancerous ascites from patients with ovarian cancer and studied it. It was found that T cells obtained from cancerous ascites could not proliferate normally after antigen stimulation, and lymphocytes could not proliferate in the acellular area of ascites. They compared cancerous ascites with normal ascites and found the difference between them is in lipid regulation, which indicates that lipid intermediates are present in ascites of ovarian cancer patients, leading to T cell dysfunction (37).

Tumor-associated bone marrow cells create an immunosuppressive microenvironment within the tumor. Leukocyte immunoglobulin-like receptor B (LILRB) family members are negative regulators of myeloid cell activation. Chen Hui-Ming et al. regulate tumor-associated myeloid cells through LILRB. In the presence of M-CSF and IL-4, LILRB2 inhibits the activation of AKT and STAT6, alters lipid/cholesterol metabolism, inhibits the infiltration of granulocyte MDSC and Treg, and significantly enhances the immune activity of T cells and promotes anti-tumor immunity (38).

Tumor cells cause metabolic reprogramming of T cells. Patsoukis Nikolaos et al. found that when PD-1 is connected, although activated T cells cannot perform glycolysis and amino acid metabolism, in this state, PD-1 can increase its fatty acids beta oxidation (FAO) rate by increasing the expression of CPT1A. At the same time, they also found that CTLA-4 can inhibit T cell glycolysis without increasing FAO, because it maintains an unactivated state and inhibits T cell antitumor immunity (39).

The conversion of cell membrane sphingomyelin (SLs) to ceramide requires the participation of acid sphingomyelinase (ASM). In cancer cells, ASM-mediated production of ceramide is essential for cell apoptosis, proliferation, and immune regulation. Kachler Katerina et al. demonstrated that in patients with non-small cell lung cancer (NSCLC), ASM activity is increased in TME and serum, and its increase can significantly inhibit Th1-mediated and CTL-mediated anti-tumor Immunity, which promotes the development of tumors (40). In addition, Batova Ayse et al. discovered that Englerin A in renal clear cell carcinoma (cc-RCC) significantly changes its lipid metabolism, and ceramide may be a mediator of Englerin A production, helping Englerin A to induce acute inflammatory response and mediate anti-tumor immunity (41). FA are important components of cell membranes, signal molecules, and bioenergy substrates. Accompanying the functional and metabolic changes that occur during the activation and differentiation of CD8^+^ T cells, fatty acid metabolism will also have corresponding changes. Lee JangEun et al. found that the tumorigenesis process is often accompanied by the loss of T cell-specific acetyl-CoA carboxylase 1 (ACC1). ACC1 catalyzes the conversion of acetyl-CoA to malonyl-CoA, which is a carbon donor in the process of long-chain fatty acid synthesis. Thus the lack of ACC1 will cause serious defects in CD8^+^T cells and promote tumor immune escape (42).

CARs are widely used clinically to redirect T cells to the cytotoxicity of cancer cells. However, the CAR’s co-stimulatory domain that affects CAR-T cell persistence and effector capacity is still uncertain. Kawalekar Omkar U et al. reported the effect of co-receptor CD28 and 4-1BB signaling domains on the metabolic characteristics of human CAR-T cells. Among them, 4-1BB increases the fatty acid oxidation of CD8^+^ T cells and mitochondrial biogenesis to promote its growth, while CD28 enhances the glycolysis of CAR-T cells, which provides a theoretical basis for the future application of CAR-T cell therapy (43).

### Dendritic Cells and Fatty Acid Metabolism

Fatty acid metabolism connects innate and adaptive immune responses by regulating DC function. In addition, DC cells are necessary to initiate and maintain T-cell-dependent anti-tumor immunity. Tumor cells usually evade immunosuppression by weakening normal DC function. Cubillos-Ruiz Juan R et al. found that the ER stress response factor XBP1 can directly promote tumor growth. In ovarian cancer, XBP1, which is promoted by lipid peroxidation byproducts, is activated to induce tumor-associated DC (tDC) to initiate triglyceride biosynthesis procedures, leading to abnormal lipid accumulation and subsequently inhibit tDC’s ability to support anti-tumor T cells, thereby achieving the purpose of inactivating anti-tumor immunity and promoting tumor development (44).

Fatty acid synthase (FASN), a key metabolic enzyme for fatty acid synthesis, can directly promote tumor proliferation and metastasis. In the study by Jiang Li et al., it was described that the later the clinical stage of ovarian cancer, the more obvious the increase of FASN expression is, and it is related to the state of immune suppression. They proposed that the intrinsic FASN of tumor cells promotes the development of ovarian cancer by weakening anti-tumor immunity, and found that the activation of FASN in cancer cells can lead to an increase in lipid concentration in TME, and the accumulation of abnormal lipids inhibits the ability of tumor infiltration DC (TIDC) to support anti-tumor T cells. DC fail to present antigens and primary T cell is disability when they are cultured in the ascites of ovarian cancer patients with activated FASN, which further support this view. In short, ovarian cancer endogenous FASN can induce tumor cells’ immune escape through lipid accumulation in TIDC and subsequent T cell rejection and dysfunction (45).

### NK Cells and Fatty Acid Metabolism

**FIGURE 2:**
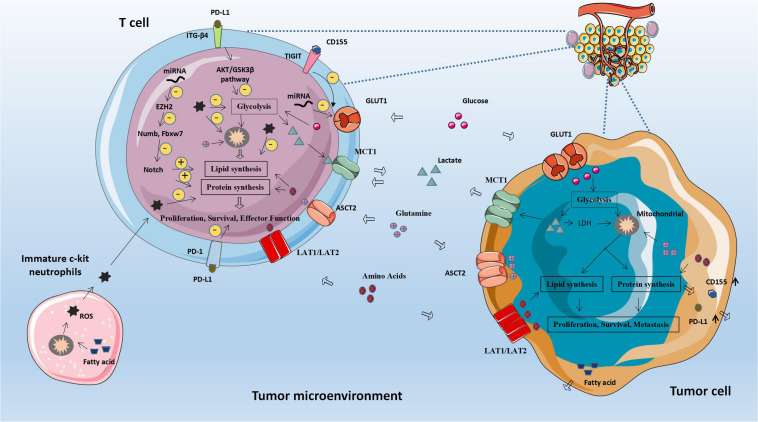
Abnormal fatty acid metabolism and tumors. The abnormal metabolism of lipids and lipid-related molecules in tumor cells will affect the metabolic level of immune cells, leading to immune cell dysfunction, achieving immune evasion and weakening anti-tumor immunity. CPT1A, Carnitine palmitoyltransferase 1A; ACC1, Acetyl-CoA carboxylase 1; ASM, Acid sphingomyelinase; MCP-1, Monocyte chemoattractant protein 1; FASN, Fatty acid synthase.

In some cancers, more than 49% is attributed to obesity, which may because of excessive production of hormones, adipokines and insulin. Cytotoxic immune cells are essential for immunodetection. Michelet Xavier et al. proposed that obesity-induced peroxisome proliferator-activated receptor (PPAR) drive lipid accumulation in NK cells and then leads to impaired cell metabolism and transport, which weakens the antitumor response of NK cells (46). Therefore, the abnormal metabolism of lipids and lipid-related molecules in tumor cells will affect the metabolic level of immune cells, leading to immune cell dysfunction, achieving immune evasion and weakening anti-tumor immunity (Figure 2 and Table 2).

**TABLE 2:** Fatty acid metabolism and tumor immunity.

Molecules/drugs	Status	Major effects	Pathway	Tumor types	Type of Immune cells	References
PD-L1	Up	Reduces cytokine secretion by activated CTLs	Up-regulating fatty acid oxidation through increased expression of CPT-I	–	T cells	(39)
Fenofibrate		Induce T cells to engage fatty acid catabolism	PPARa	–	T cells	(96)
CD47	Up	Avoidance of metabolic changes in central carbon of macrophages	CpG oligodeoxynucleotide	–	Macrophages	(33)
GRP78	–	Increased macrophage infiltration	Increase MCP-1 serum levels and decrease CD47 expression	–	Macrophages	(32)
LILRB	–	Enhance the immune activity of T cells	Inhibits AKT and STAT6 activation	–	T cells	(38)
ASM	Up	Inhibition of Th1-mediated and cytotoxic T cell-mediated anti-tumor immunity		NSCLC		(40)
XBP1	–	Inhibition of tDC support against tumor T cells	Induction of tDC initiates triglyceride biosynthesis program	Ovarian cancer	DC	(44)
ACC1	Down	Severe defects in CD8 T cells	Catalyzes the conversion of acetyl-CoA to malonyl-CoA	–	CD8 T cells	(42)
CD28 4-1BB	–	Promote the growth of CD8 T cells	Increases fatty acid oxidation and mitochondrial biogenesis in CD8 T cells	–	CD8 T cells	(43)
PPAR	–	NK cell metabolism and transport disorders	Drives NK cell lipid accumulation	–	NK cells	(46)
FASN	Up	Inhibits the ability of TIDC-supported anti-tumor T cells	Causes increased lipid concentration in TME	–	T cells	(45)

## Amino Acid Metabolism and Tumor Immunity

T lymphocyte metabolism changes with its functional status (47). For resting T cells, only low oxidative phosphorylation is required to maintain their normal life activities. When T cells are activated, they must proliferate in large numbers to produce sufficient effector cells. At this time, the metabolic requirements of T cells are significantly increased, and the main pathway of energy production also changes from low levels of oxidative phosphorylation to high levels of glycolysis and amino acid metabolism to support the synthesis of nucleotide and lipid, which is necessary for its growth and proliferation (48).

During the tumorigenesis process, due to many physiological changes, some new immune checkpoints will be generated, and these physiological changes can reprogram the inflammation, immune and metabolic processes in malignant lesions and local lymphoid tissues, thus constituting a TME that suppresses immune effects. Elevated catabolism of tryptophan (Trp) and arginine (Arg) is a common TME marker in the clinical manifestations of cancer. Indoleamine 2,3 dioxygenase (IDO) and arginase 1 (ARG1) break down Trp and Arg, respectively, and respond to inflammatory signals including interferon and TGFβ cytokines (49). Amino acid degradation reactions mediated by IDO and ARG1 have become key factors in regulating tumor-induced immune tolerance. Tryptophan degrading enzymes and arginine degrading enzymes expressed by tumors and tumor infiltrating cells can effectively impede cancer-specific immune responses (50).

Originally, tryptophan dioxygenase (TDO) was considered to be the only enzyme capable of metabolizing l-tryptophan, which is an essential amino acid. However, IDO was later found in rabbits and proved to be able to metabolize both l-tryptophan and d-tryptophan at the same time (51). IDO is associated with a variety of immune diseases such as cancer, allergies, autoimmunity, and inflammation. In some tumors, there is abundant lymphocyte infiltration around IDO1-expressing tumor cells, which means that IDO expression may be the result of IFN-γ expression and drug resistance mechanisms. In other cancers, IDO1 expression is constitutive, while tumor cells expressing IDO1 are surrounded by fewer lymphocytes (52).

Indoleamine 2,3 dioxygenase 1 and TDO contain a group of enzymes that are required for the first step in catalyzing the decomposition of Trp into kynurenine (Kyn), and also the rate-limiting step of this reaction. The Kyn generated by this reaction is further converted into the high-energy substrate NAD (+) And ATP, which energize cell activity, and in tumor cells, IDO activity significantly increases, which causes the lack of Trp in TME and the accumulation of downstream product Kyn, while the depletion of Trp and the excessive accumulation of Kyn induce effects T cell apoptosis, dysfunction and induction of immunosuppressive regulatory T cells. In short, tryptophan metabolism is essential for cell proliferation, inflammatory response and immune regulation, and accelerated tryptophan breakdown promotes immune escape of tumors (53–55).

As described previously, the catabolism of arginine is significantly increased in tumor tissues, which can lead to a decrease in the intracellular L-arginine concentration. L-arginine levels are closely related to the activation and maintenance of T cells. High levels of L-arginine can promote the metabolism of T cells from glycolysis to oxidative phosphorylation, thereby activating T cells and promoting the production of central memory cells. In contrast, low L-arginine concentrations in tumor tissues hinder T cell activation and inhibit T cell function (56). And myeloid-derived suppressor cells (MDSCs) are heterogeneous populations of immature cells that expand during the inflammatory process caused by tumor tissues and then increase the metabolism of l-arginine (l-Arg) by ARG1 and nitric oxide synthase 2 (NOS2), thereby suppressing the immune capacity of T cells (57).

Tumors can create a microenvironment that interferes with metabolism and effector functions of T cell to escape immune control. Ovarian cancer tissue can induce ER stress and activate the IRE1α-XBP1 arm of T cells involved in protein expansion response, thereby controlling the mitochondrial function and antitumor ability of T cells. The study of T cells isolated from ovarian cancer patients found that up-regulation of XBP1 would reduce the ability of T cells to infiltrate tumor tissues and reduce the expression of IFNG mRNA. Tumor tissue can inhibit the uptake of glucose in T cells and cause defects in intracellular N-catenin glycosylation, which triggers the activation of IRE1α-XBP1. The induction of XBP1 regulates the abundance of glutamine carriers on T cell membranes, which prevents glutamine from entering mitochondria under conditions of glucose deficiency. And glutamine is necessary for mitochondrial respiration. Thus XBP1 ultimately inhibits the activity of mitochondria and hinders the production of IFN-γ (58). One of the hallmarks of renal clear cell carcinoma is glutamine addiction. Excessive consumption of glutamine caused by glutamine addiction can result in local deprivation of glutamine outside the cell, which can induce macrophages to secrete IL23 through activation of hypoxia-inducible factor 1α (HIF1α), and IL23 activates the proliferation of Treg cells, promotes the expression of IL10 and TGF β, thereby inhibiting cytotoxic lymphocytes from killing tumor cells and coordinating immune escape (59).

It was reported that DC-HIL binds to syndecan-4 on effector T cells and produces an inhibitory effect on T cells. The concentrations of IL-1β and IFN-γ in melanoma tissues are higher than those in normal tissues. Both of them can induce the expression of DC-HIL through tumor-infiltrating CD11b (+) Gr1 (+) cells. DC-HIL can promote intracellular immune receptor tyrosine activation phosphorylation, induce intracellular expression of IFN-γ and nitric oxide synthase, and further inhibit T cell function (60).

**FIGURE 3:**
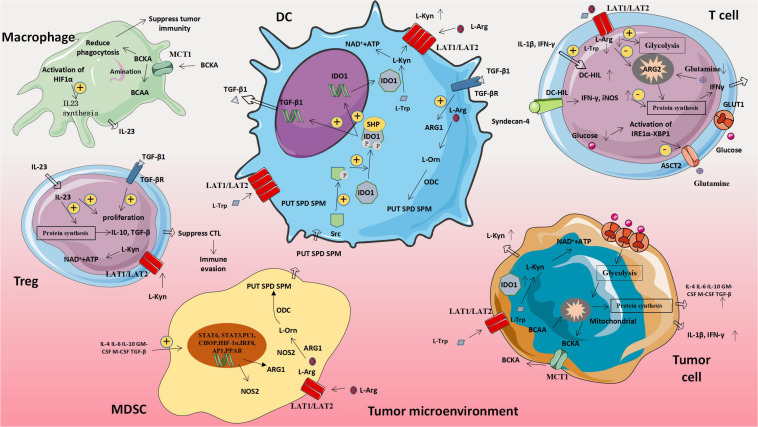
Amino acid-related metabolic abnormalities and tumors. The metabolism of abnormal amino acids and amino acids related moleculars in tumor cells will affect the expression of metabolism-related genes in immune cells, thus leading to the increased expression of molecules that promote tumor proliferation and weaken anti-tumor immune effects. MDSC, Myeloid-derived suppressor cell; ARG1, Arginase 1; L-Orn, L-Ornithine; L-Arg, L-Arginine; L-Trp, L-Tryptophan; L-Kyn, L-Kynurenine; PUT, Putrescine; SPD, Spermidine; SPM, Spermine; ODC, Ornithine decarboxylase; NOS2, Nitric oxide synthase 2; BCAA, Branched chain amino acid; and BCKA, Branched keto acid.

L-amino acid oxidase (LAAO) is a flavin adenine dinucleotide-dependent enzyme, the most typical of which is IL4I1, which mainly oxidizes 1-phenylalanine. IL4I1 can inhibit T cell proliferation and cytokine production by limiting the ability of T cells to respond to the stimulation of cloned receptors. In addition, IL4I1 can also promote the differentiation of naive CD4^+^T cells into regulatory T cells, thereby suppressing effector T cells’ function (61). The metabolism of abnormal amino acids and amino acids related molecules in tumor cells will affect the expression of metabolism-related genes in immune cells, thus leading to the increased expression of molecules that promote tumor proliferation and weaken anti-tumor immune effects (Figure 3 and Table 3).

**TABLE 3:** Amino acid metabolism and tumor immunity.

Molecules/drugs	Status	Major effects	Pathway	Tumor types	Type of Immune cells	References
SLC1A5	Up	Impairs proliferation and the release of INF-g by activated murine splenic T cells	Reduce extracellular glutamine concentration	–	T cells	(97)
IDO	Up	Induce cell cycle arrest, anergy and apoptosis in T cells	Catalyze the conversion of tryptophan to kynurenine	–	T cells	(98)
Rapamycin	–	Activate glutamate dehydrogenase and stimulate the glutamine mitochondrial uptake; favor the generation of memory-like T cells	mTORC1	–	T cells	(99)
ASCT2	Down	Block the induction of T helper 1 (Th1) and Th17 cells	Reduce T-cell glutamine uptake	–	T cells	(100)
Myc	Mutate	Reduced extracellular glutamine concentration	Tumor cell’s high glutamine uptake	–	T cells	(101)
IL-1β IFN-γ	Up	Inhibit T cell function	Induce DC-HIL expression by tumor infiltrating CD11b (+) Gr1 (+) cells	–	T cells	(60)
MDSC	Up	Suppress T-cell immunity	Increased l-Arg metabolism through arginase 1 and NOS2	–	T cells	(57)
L-arginine	Down	Suppress T-cell function	Prevents T cell metabolism from glycolysis to oxidative phosphorylation	–	T cells	(56)

## Drug Resistance and Metabolism

Many drugs were originally designed to inhibit certain specific targets, and later experiments have shown that their effects are related to metabolism. And metabolic reprogramming may also be a way for cancer cells to escape from the selective inhibition of target drugs and become resistant.

### Amino Acid Metabolism and Drug Resistance

Immunotherapy has become the main means of fighting cancer. However, for breast cancer, its responsiveness to immunotherapy is low, so it is necessary to find effective strategies to overcome the resistance of immunotherapy. Among them, triple negative breast cancer (TNBC) is the most difficult Type, which is largely related to the plasticity of tumor cells and the persistence of cancer stem cells (CSC). Conventional chemotherapy methods have enriched CSC, leading to drug resistance and disease recurrence. Showalter Loral E et al. found that the expression of histone lysine-specific demethylase 1 (LSD1) is inversely proportional to chemokine CCL5 levels that attract CTLs, while the use of LSD1 inhibitors combined with PD-1 antibodies can significantly increase the infiltration of CD8 ^+^ T cells and reduce the tolerance of TNBC to chemotherapy drugs (62, 63). In addition, Chen Weilong et al. found that during the taxane-containing chemotherapy, the CC motif chemokine ligand 20 (CCL20) was significantly elevated, which is activated by protein kinase Cζ (PKCζ) or p38 mitogen Protein kinase (MAPK) -mediated NF-κB activation to promote self-renewal and maintenance of CSC or breast cancer stem-like cells, while NF-κB activation increases CCL20 expression, forming a positive feedback loop between the NF-κB and CCL20 pathways, providing continuous momentum for chemo-resistance in breast cancer cells (64–66). Yu Shiyi et al. found that heat shock protein 90 (HSP90) and histone deacetylase 6 (HDAC6) are promising anticancer drug targets. Anti-HDAC6 can effectively improve the resistance of TNBC to tamoxifen (67, 68). In HER2 (+) breast cancer, the targeted anticancer drug lapatinib can directly interfere with HER-2 signaling to inhibit cancer cell proliferation. When patients show resistance to lapatinib, combined application of Th1 cytokines can greatly inhibit the metabolism of cancer cells, enhance the metabolic inhibition induced by lapatinib, and almost abolish drug resistance (62).

Over-activation of tyrosine kinase (JAK) and related transcription factors STAT3 and STAT5 in the cytoplasm of tumor cells can be found in most of the malignant tumors. JAK/STAT3 signal transduction has the ability to suppress tumor immunity, and the application of JAK inhibitors in colon cancer can suppress sporadic colon cancer caused by mutations in APC inhibitory genes, and it may also provide treatment opportunities for other oncogenes with resistance (69). Inhibitory LILRB1-5 signal through their immunoreceptor tyrosine-based inhibitory motif (ITIM) in their intracellular domain and recruit the phosphatase protein tyrosine phosphatase (PTPN), which can directly regulate the development, drug resistance and recurrence of cancer, and when PTPN is missing, it can induce the resistance of CD 8 T cells to the inhibitory effect of TGFβ, which can improve the sensitivity of tumor treatment (70–72). Tyrosine kinase inhibitor (EGFR-TKI) has achieved good clinical results in the treatment of patients with NSCLC. However, recent clinical studies have shown that Adenine nucleotide translocase-2 (ANT2) can promote tumor cells to EGFR -TKI resistance, in addition, the generation of EGFR-TKI function involves the ErbB receptor pathway, however, in cancer tissues that are resistant to EGFR-TKI, ErbB receptor-independent oncogenic pathway genes are overexpressed and the biosynthesis of partially inflammatory cytokines is too active, so it is speculated that using EGFR-TKI with antibody inhibitors may have a better chance of avoiding drug resistance (73, 74). In addition to being able to escape the cytotoxic effects of chemotherapy, multidrug-resistant cancer cells bypass the anti-cancer drug-induced immunogenicity. In their research, it was found that intracellular IDO1 activity and expression are both higher, JAK1/STAT1 and JAK1/STAT3 signaling have higher basic activities, and the STAT3 inhibitor PIAS3 is down-regulated. Campia Ivana et al. proposed that due to Constitutive activation of JAK/STAT/IDO1 axis, multidrug-resistant cells have a stronger degree of immunosuppression than chemically sensitive cells, leading to chemical and immune evasion, so destroying this axis may significantly improve chemoimmunotherapy regimens for tumors’ drug-resistant (75).

### Glucose Metabolism and Drug Resistance

In glucose metabolism, the TCA cycle is considered to be an internal clean metabolic pathway, which is essential for energy production and biosynthetic intermediates. Changes in the TCA cycle play a key role in tumorigenesis and inflammation, and have a certain relationship with tumor drug resistance mechanisms (76). Among the Warburg effects caused by tumors, HIF1 is the main hypoxia-induced transcription factor, which promotes dissociation between glycolysis and the TCA cycle, restricts the effective production of ATP and citric acid to prevent glycolysis. And the Warburg effect causes the accumulation of lactic acid, which will promote tumor progression, inhibit T cell function, and make it resist to some anti-tumor therapies. Therefore, the design of new anti-tumor drugs against HIF1 can solve the problem of tumor resistance to chemotherapeutics to a certain extent (77–80).

### Fatty Acid Metabolism and Drug Resistance

Sphingomyelin is a necessary class of biologically active lipids, which are key components of cell membranes and are involved in cell differentiation, apoptosis, aging and other processes. Cancer cells usually show increased growth effects and escape from the cell death process. Studies have shown that enzymes involved in SL synthesis and catabolism can change in cancer cells, allowing cancer cells to acquire resistance properties (81).

Epithelial to mesenchymal transition (EMT) is a manifestation of malignant tumors, characterized by invasion and metastasis, enhanced chemical resistance, and escape from host immunity. We found that the inhibitory effect of PI3K-AKT-mTOR signal significantly reduced glycolysis and fatty acid oxidation and inhibited tumor growth, while phospholipase A2 (PLA2) can mobilize lysophospholipids and free FA to maintain fatty acid oxidation and oxidation phosphorylation, which reduces the inhibitory effect of PI3K-AKT-mTOR signaling, provides a new strategy for cancer treatment. In addition, the mTOR inhibitor PP242 can restore EMT, increase the expression of PD-L1 in cancer cells, and restore the sensitivity of cancer cells to chemical drugs (82–87). As shown in Table 4, these molecules are linked with drug resistance and metabolism.

**TABLE 4:** Drug resistance and metabolism.

Molecules/drugs	Status	Major effects	Pathway	Tumor types	Type of Immune cells	References
LSD1	Up	Decreases TNBC tolerance to chemotherapy drugs	LSD1 inhibitor combined with PD-1 antibody can significantly increase CD8 ^+^ T cell infiltration	TNBC	CD8 ^+^ T cells	(62)
CCL20	Up	Promote self-renewal of CSC or breast cancer stem-like cells	PKCζ/MAPK	TNBC	–	(66)
JAK	Up	Promote colon cancer tolerance to chemotherapy drugs	APC	colon cancer	–	(69)
PTPN	Down	Improve the sensitivity of tumor treatment	Induces resistance of CD8 ^+^ T cells to TGFβ inhibition	–	CD8 ^+^ T cells	(70)
ANT2	–	Promote tumor cell resistance to EGFR-TKI	–	NSCLC	–	(73)
IDO1	Up	Causes chemical and immune evasion	JAK1/STAT1 JAK1/STAT3	–	–	(75)
HIF1	Up	Promote tumor resistance to chemotherapy drugs	Promotes dissociation between glycolysis and the TCA cycle	–	–	(77)
PLA2	–	Restore cancer cells’ sensitivity to chemicals	PI3K-AKT-mTOR	–	–	(82)

## Conclusion and Future Perspectives

The occurrence and development of tumors are accompanied by metabolic reprogramming. The effect of metabolism on immunity cannot be ignored. In this review, we provide a comprehensive overview of molecular, cellular, and microenvironmental mechanisms that together help understand how tumors can alter their own metabolism to achieve immune escape. Changes in the metabolism of sugar, fat, and amino acids affect immune cells. In order to use these metabolic characteristics to increase the efficacy of existing therapies, reduce the drug resistance of tumors or discover new treatments, many studies have been conducted in the field of tumor metabolic reprogramming. These studies describe the latest developments in the field and predict the problems that need to be solved.

## Author Contributions

FW, YC, and LW were responsible for gathering information of the related research and designing the review. WeZ, WuZ, and QW were responsible for language editing. WT, XP, and HC has contributed to information interpretation, editing, and critical revision of the manuscript. All authors read and approved the final manuscript.

## Conflict of Interest

The authors declare that the research was conducted in the absence of any commercial or financial relationships that could be construed as a potential conflict of interest.

## Abbreviations

ACC1: acetyl-CoA carboxylase 1
ARG: arginase
Arg: arginine
ARG1: arginase 1
ASM: acid sphingomyelinase
cc-RCC: renal clear cell carcinoma
CIC: citric acid carrier
CSC: cancer stem cells
CTL: cytotoxic T cells
DC: Dendritic cells
EMT: Epithelial to mesenchymal transition
ER: endoplasmic reticulum
FA: fatty acids
FAO: fatty acids beta oxidation
FASN: Fatty acid synthase
GLS: glutaminase
Glut-1: glucose transporter 1
GRP78: glucose-regulated protein 78
HDAC6: histone deacetylase 6
HIF1 α: hypoxia-inducible factor 1 α
HSP90: heat shock protein 90
IDO: Indoleamine 2,3 dioxygenase
ITGB4: integrin β 4
ITIM: immunoreceptor tyrosine-based inhibitory motif
JAK: tyrosine kinase
Kyn: kynurenine
LAAO: L-amino acid oxidase
l-Arg: l-arginine
LILRB: Leukocyte immunoglobulin-like receptor B
LILRB1: leukocyte immunoglobulin-like receptors B1
LSD1: lysine-specific demethylase 1
MAPK: mitogen Protein kinase
MDSCs: myeloid-derived suppressor cells
NOS2: nitric oxide synthase 2
NSCLC: non-small cell lung cancer
PD-1: programmed cell death protein 1
PKC ζ: protein kinase C ζ
PLA2: phospholipase A2
PPAR: peroxisome proliferator-activated receptor
PPP: Pentose Phosphate Pathway
PTPN: phosphatase protein tyrosine phosphatase
ROS: reactive oxygen species
SL: Sphingomyelin
TCA cycle: tricarboxylic acid cycle
tDC: tumor-associated DC
TDO: tryptophan dioxygenase
TIDC: tumor infiltration DC
Tm: memory T cells
TME: tumor microenvironment
TNBC: triple negative breast cancer
Treg: regulatory T cells
Trp: tryptophan
VHL: von Hippel-Lindau.

## References

[1] Lawrence MS Stojanov P Polak P Kryukov GV Cibulskis K Sivachenko A Mutational heterogeneity in cancer and the search for new cancer-associated genes. *Nature.* (2013) 499:214–8. 23770567 10.1038/nature12213 PMC3919509

[2] Hanahan D Weinberg RA. Hallmarks of cancer: the next generation. *Cell.* (2011) 144:646–74. 10.1016/j.cell.2011.02.013 21376230

[3] Biswas SK Mantovani A. Macrophage plasticity and interaction with lymphocyte subsets: cancer as a paradigm. *Nat Immunol.* (2010) 11:889–96. 20856220 10.1038/ni.1937

[4] Ward PS Thompson CB. Metabolic reprogramming: a cancer hallmark even warburg did not anticipate. *Cancer Cell.* (2012) 21:297–308. 10.1016/j.ccr.2012.02.014 22439925 PMC3311998

[5] Cantor JR Sabatini DM. Cancer cell metabolism: one hallmark, many faces. *Cancer Discov.* (2012) 2:881–98. 10.1158/2159-8290.cd-12-0345 23009760 PMC3491070

[6] Liberti MV Locasale JW. The warburg effect: how does it benefit cancer cells? *Trends Biochem Sci.* (2016) 41:211–8. 10.1016/j.tibs.2015.12.001 26778478 PMC4783224

[7] Lunt SY Vander Heiden MG. Aerobic glycolysis: meeting the metabolic requirements of cell proliferation. *Annu Rev Cell Dev Biol.* (2011) 27:441–64. 10.1146/annurev-cellbio-092910-154237 21985671

[8] de la Cruz-López KG Castro-Muñoz LJ Reyes-Hernández DO García-Carrancá A Manzo-Merino J. Lactate in the regulation of tumor microenvironment and therapeutic approaches. *Front Oncol.* (2019) 9:1143. 10.3389/fonc.2019.01143 31737570 PMC6839026

[9] Garcia-Cao I Song MS Hobbs RM Laurent G Giorgi C de Boer VC Systemic elevation of PTEN induces a tumor-suppressive metabolic state. *Cell.* (2012) 149:49–62. 10.1016/j.cell.2012.02.030 22401813 PMC3319228

[10] Chan DA Sutphin PD Nguyen P Turcotte S Lai EW Banh A Targeting GLUT1 and the warburg effect in renal cell carcinoma by chemical synthetic lethality. *Sci Transl Med.* (2011) 3:94ra70. 10.1126/scitranslmed.3002394 21813754 PMC3683134

[11] Wise DR DeBerardinis RJ Mancuso A Sayed N Zhang XY Pfeiffer HK Myc regulates a transcriptional program that stimulates mitochondrial glutaminolysis and leads to glutamine addiction. *Proc Natl Acad Sci USA.* (2008) 105:18782–7. 10.1073/pnas.0810199105 19033189 PMC2596212

[12] Huypens PR Huang M Joseph JW. Overcoming the spatial barriers of the stimulus secretion cascade in pancreatic beta-cells. *Islets.* (2012) 4:1–116. 10.4161/isl.18338 22143007

[13] Kishton RJ Sukumar M Restifo NP. Metabolic regulation of T cell longevity and function in tumor immunotherapy. *Cell Metab.* (2017) 26:94–109. 10.1016/j.cmet.2017.06.016 28683298 PMC5543711

[14] Siska PJ Rathmell JC. T cell metabolic fitness in antitumor immunity. *Trends Immunol.* (2015) 36:257–64. 10.1016/j.it.2015.02.007 25773310 PMC4393792

[15] Geltink RIK Kyle RL Pearce EL. Unraveling the complex interplay between T cell metabolism and function. *Annu Rev Immunol.* (2018) 36:461–88. 10.1146/annurev-immunol-042617-053019 29677474 PMC6323527

[16] Buck MD O’Sullivan D Pearce EL. T cell metabolism drives immunity. *J Exp Med.* (2015) 212:1345–60. 10.1084/jem.20151159 26261266 PMC4548052

[17] Ohashi T Akazawa T Aoki M Kuze B Mizuta K Ito Y Dichloroacetate improves immune dysfunction caused by tumor-secreted lactic acid and increases antitumor immunoreactivity. *Int J Cancer.* (2013) 133:1107–18. 10.1002/ijc.28114 23420584

[18] Keir ME Butte MJ Freeman GJ Sharpe AH. PD-1 and its ligands in tolerance and immunity. *Annu Rev Immunol.* (2008) 26:677–704. 10.1146/annurev.immunol.26.021607.090331 18173375 PMC10637733

[19] Cai G Karni A Oliveira EM Weiner HL Hafler DA Freeman GJ. PD-1 ligands, negative regulators for activation of naive, memory, and recently activated human CD4+ T cells. *Cell Immunol.* (2004) 230:89–98. 10.1016/j.cellimm.2004.09.004 15598424

[20] Wang S Li J Xie J Liu F Duan Y Wu Y Programmed death ligand 1 promotes lymph node metastasis and glucose metabolism in cervical cancer by activating integrin beta4/SNAI1/SIRT3 signaling pathway. *Oncogene.* (2018) 37:4164–80. 10.1038/s41388-018-0252-x 29706653

[21] Siska PJ van der Windt GJ Kishton RJ Cohen S Eisner W MacIver NJ Suppression of Glut1 and glucose metabolism by decreased Akt/mTORC1 signaling drives T cell impairment in B cell leukemia. *J Immunol.* (2016) 197:2532–40. 10.4049/jimmunol.1502464 27511728 PMC5010978

[22] Polesso F Weinberg AD Moran AE. Late-stage tumor regression after PD-L1 blockade plus a concurrent OX40 agonist. *Cancer Immunol Res.* (2019) 7:269–81. 10.1158/2326-6066.cir-18-0222 30563828

[23] Gracias DT Stelekati E Hope JL Boesteanu AC Doering TA Norton J The microRNA miR-155 controls CD8(+) T cell responses by regulating interferon signaling. *Nat Immunol.* (2013) 14:593–602. 10.1038/ni.2576 23603793 PMC3664306

[24] Zhang T Zhang Z Li F Ping Y Qin G Zhang C miR-143 regulates memory T cell differentiation by reprogramming T cell metabolism. *J Immunol.* (2018) 201:2165–75. 10.4049/jimmunol.1800230 30150287

[25] Zhao E Maj T Kryczek I Li W Wu K Zhao L Cancer mediates effector T cell dysfunction by targeting microRNAs and EZH2 via glycolysis restriction. *Nat Immunol.* (2016) 17:95–103. 10.1038/ni.3313 26523864 PMC4684796

[26] Rice CM Davies LC Subleski JJ Maio N Gonzalez-Cotto M Andrews C Tumour-elicited neutrophils engage mitochondrial metabolism to circumvent nutrient limitations and maintain immune suppression. *Nat Commun.* (2018) 9:5099. 10.1038/s41467-018-07505-2 PMC6269473 30504842

[27] He W Zhang H Han F Chen X Lin R Wang W CD155T/TIGIT signaling regulates CD8(+) T-cell metabolism and promotes tumor progression in human gastric cancer. *Cancer Res.* (2017) 77:6375–88. 10.1158/0008-5472.can-17-0381 28883004

[28] Li L Liu X Sanders KL Edwards JL Ye J Si F TLR8-mediated metabolic control of human treg function: a mechanistic target for cancer immunotherapy. *Cell Metab.* (2019) 29:103–23.e105. 30344014 10.1016/j.cmet.2018.09.020 PMC7050437

[29] Marijt KA Sluijter M Blijleven L Tolmeijer SH Scheeren FA van der Burg SH Metabolic stress in cancer cells induces immune escape through a PI3K-dependent blockade of IFNgamma receptor signaling. *J Immunother Cancer.* (2019) 7:152. 10.1186/s40425-019-0627-8 PMC6567539 31196219

[30] Catalán E Charni S Jaime P Aguiló JI Enríquez JA Naval J MHC-I modulation due to changes in tumor cell metabolism regulates tumor sensitivity to CTL and NK cells. *Oncoimmunology.* (2015) 4:e985924. 10.4161/2162402x.2014.985924 25949869 PMC4368123

[31] Ma R Ji T Zhang H Dong W Chen X Xu P A Pck1-directed glycogen metabolic program regulates formation and maintenance of memory CD8(+) T cells. *Nat Cell Biol.* (2018) 20:21–7. 10.1038/s41556-017-0002-2 29230018

[32] Cook KL Soto-Pantoja DR Clarke PA Cruz MI Zwart A Wärri A Endoplasmic reticulum stress protein GRP78 modulates lipid metabolism to control drug sensitivity and antitumor immunity in breast cancer. *Cancer Res.* (2016) 76:5657–70. 10.1158/0008-5472.can-15-2616 27698188 PMC5117832

[33] Liu M O’Connor RS Trefely S Graham K Snyder NW Beatty GL Metabolic rewiring of macrophages by CpG potentiates clearance of cancer cells and overcomes tumor-expressed CD47-mediated ‘don’t-eat-me’ signal. *Nat Immunol.* (2019) 20:265–75. 10.1038/s41590-018-0292-y 30664738 PMC6380920

[34] MacIver NJ Michalek RD Rathmell JC. Metabolic regulation of T lymphocytes. *Annu Rev Immunol.* (2013) 31:259–83. 10.1146/annurev-immunol-032712-095956 23298210 PMC3606674

[35] Wang C Yosef N Gaublomme J Wu C Lee Y Clish CB CD5L/AIM regulates lipid biosynthesis and restrains Th17 cell pathogenicity. *Cell.* (2015) 163:1413–27. 10.1016/j.cell.2015.10.068 26607793 PMC4671820

[36] van der Windt GJ Everts B Chang CH Curtis JD Freitas TC Amiel E Mitochondrial respiratory capacity is a critical regulator of CD8+ T cell memory development. *Immunity.* (2012) 36:68–78. 10.1016/j.immuni.2011.12.007 22206904 PMC3269311

[37] Wefers C Duiveman-de Boer T Zusterzeel PLM Massuger LFAG Fuchs D Torensma R Different lipid regulation in ovarian cancer: inhibition of the immune system. *Int J Mol Sci.* (2018) 19:273. 10.3390/ijms19010273 29342108 PMC5796219

[38] Chen HM van der Touw W Wang YS Kang K Mai S Zhang J Blocking immunoinhibitory receptor LILRB2 reprograms tumor-associated myeloid cells and promotes antitumor immunity. *J Clin Invest.* (2018) 128:5647–62. 10.1172/jci97570 30352428 PMC6264729

[39] Patsoukis N Bardhan K Chatterjee P Sari D Liu B Bell LN PD-1 alters T-cell metabolic reprogramming by inhibiting glycolysis and promoting lipolysis and fatty acid oxidation. *Nat Commun.* (2015) 6:6692. 10.1038/ncomms7692 PMC4389235 25809635

[40] Kachler K Bailer M Heim L Schumacher F Reichel M Holzinger CD Enhanced acid sphingomyelinase activity drives immune evasion and tumor growth in non-small cell lung carcinoma. *Cancer Res.* (2017) 77:5963–76. 10.1158/0008-5472.can-16-3313 28883000

[41] Batova A Altomare D Creek KE Naviaux RK Wang L Li K Englerin A induces an acute inflammatory response and reveals lipid metabolism and ER stress as targetable vulnerabilities in renal cell carcinoma. *PLoS One.* (2017) 12:e0172632. 10.1371/journal.pone.0172632 28296891 PMC5351975

[42] Lee J Walsh MC Hoehn KL James DE Wherry EJ Choi Y Regulator of fatty acid metabolism, acetyl coenzyme a carboxylase 1, controls T cell immunity. *J Immunol.* (2014) 192:3190–9. 10.4049/jimmunol.1302985 24567531 PMC3965631

[43] Kawalekar OU O’Connor RS Fraietta JA Guo L McGettigan SE Posey AD Jr. Distinct signaling of coreceptors regulates specific metabolism pathways and impacts memory development in CAR T cells. *Immunity.* (2016) 44:380–90. 10.1016/j.immuni.2016.01.021 26885860 PMC13498396

[44] Cubillos-Ruiz JR Silberman PC Rutkowski MR Chopra S Perales-Puchalt A Song M ER stress sensor XBP1 controls anti-tumor immunity by disrupting dendritic cell homeostasis. *Cell.* (2015) 161:1527–38. 10.1016/j.cell.2015.05.025 26073941 PMC4580135

[45] Jiang L Fang X Wang H Li D Wang X. Ovarian cancer-intrinsic fatty acid synthase prevents anti-tumor immunity by disrupting tumor-infiltrating dendritic cells. *Front Immunol.* (2018) 9:2927. 10.3389/fimmu.2018.02927 30619288 PMC6302125

[46] Michelet X Dyck L Hogan A Loftus RM Duquette D Wei K Metabolic reprogramming of natural killer cells in obesity limits antitumor responses. *Nat Immunol.* (2018) 19:1330–40. 10.1038/s41590-018-0251-7 30420624

[47] Pearce EL Poffenberger MC Chang CH Jones RG. Fueling immunity: insights into metabolism and lymphocyte function. *Science.* (2013) 342:1242454. 10.1126/science.1242454 24115444 PMC4486656

[48] Gerriets VA Kishton RJ Nichols AG Macintyre AN Inoue M Ilkayeva O Metabolic programming and PDHK1 control CD4+ T cell subsets and inflammation. *J Clin Invest.* (2015) 125:194–207. 10.1172/jci76012 25437876 PMC4382238

[49] Lemos H Huang L Prendergast GC Mellor AL. Immune control by amino acid catabolism during tumorigenesis and therapy. *Nat Rev Cancer.* (2019) 19:162–75. 10.1038/s41568-019-0106-z 30696923

[50] Timosenko E Hadjinicolaou AV Cerundolo V. Modulation of cancer-specific immune responses by amino acid degrading enzymes. *Immunotherapy.* (2017) 9:83–97. 10.2217/imt-2016-0118 28000524

[51] Yamamoto S Hayaishi O. Tryptophan pyrrolase of rabbit intestine. D- and L-tryptophan-cleaving enzyme or enzymes. *J Biol Chem.* (1967) 242:5260–6. 6065097

[52] Uyttenhove C Pilotte L Théate I Stroobant V Colau D Parmentier N Evidence for a tumoral immune resistance mechanism based on tryptophan degradation by indoleamine 2,3-dioxygenase. *Nat Med.* (2003) 9:1269–74. 10.1038/nm934 14502282

[53] Gostner JM Becker K Überall F Fuchs D. The potential of targeting indoleamine 2,3-dioxygenase for cancer treatment. *Expert Opin Ther Targets.* (2015) 19:605–15. 10.1517/14728222.2014.995092 25684107

[54] Zhai L Spranger S Binder DC Gritsina G Lauing KL Giles FJ Molecular pathways: targeting IDO1 and other tryptophan dioxygenases for cancer immunotherapy. *Clin Cancer Res.* (2015) 21:5427–33. 10.1158/1078-0432.ccr-15-0420 26519060 PMC4681601

[55] Amobi A Qian F Lugade AA Odunsi K. Tryptophan catabolism and cancer immunotherapy targeting IDO mediated immune suppression. *Adv Exp Med Biol.* (2017) 1036:129–44. 10.1007/978-3-319-67577-0_9 29275469

[56] Geiger R Rieckmann JC Wolf T Basso C Feng Y Fuhrer T L-arginine modulates T cell metabolism and enhances survival and anti-tumor activity. *Cell.* (2016) 167:829–42.e813. 27745970 10.1016/j.cell.2016.09.031 PMC5075284

[57] Cimen Bozkus C Elzey BD Crist SA Ellies LG Ratliff TL. Expression of cationic amino acid transporter 2 is required for myeloid-derived suppressor cell-mediated control of T cell immunity. *J Immunol.* (2015) 195:5237–50. 10.4049/jimmunol.1500959 26491198 PMC4655170

[58] Song M Sandoval TA Chae CS Chopra S Tan C Rutkowski MR IRE1alpha-XBP1 controls T cell function in ovarian cancer by regulating mitochondrial activity. *Nature.* (2018) 562:423–8. 10.1038/s41586-018-0597-x 30305738 PMC6237282

[59] Fu Q Xu L Wang Y Jiang Q Liu Z Zhang J Tumor-associated macrophage-derived Interleukin-23 interlinks kidney cancer glutamine addiction with immune evasion. *Eur Urol.* (2019) 75:752–63. 10.1016/j.eururo.2018.09.030 30293904

[60] Chung JS Tamura K Cruz PD Jr. Ariizumi K. DC-HIL-expressing myelomonocytic cells are critical promoters of melanoma growth. *J Invest Dermatol.* (2014) 134:2784–94. 10.1038/jid.2014.254 24936834 PMC4199867

[61] Castellano F Molinier-Frenkel V. An overview of l-amino acid oxidase functions from bacteria to mammals: focus on the immunoregulatory phenylalanine oxidase IL4I1. *Molecules.* (2017) 22:2151. 10.3390/molecules22122151 29206151 PMC6149928

[62] Showalter LE Oechsle C Ghimirey N Steele C Czerniecki BJ Koski GK. Th1 cytokines sensitize HER-expressing breast cancer cells to lapatinib. *PLoS One.* (2019) 14:e0210209. 10.1371/journal.pone.0210209 30657766 PMC6338365

[63] Sulaiman A McGarry S Lam KM El-Sahli S Chambers J Kaczmarek S Co-inhibition of mTORC1, HDAC and ESR1alpha retards the growth of triple-negative breast cancer and suppresses cancer stem cells. *Cell Death Dis.* (2018) 9:815. 10.1038/s41419-018-0811-7 PMC6062597 30050079

[64] Chen W Qin Y Wang D Zhou L Liu Y Chen S CCL20 triggered by chemotherapy hinders the therapeutic efficacy of breast cancer. *PLoS Biol.* (2018) 16:e2005869. 10.1371/journal.pbio.2005869 30052635 PMC6082578

[65] Zeligs KP Neuman MK Annunziata CM. Molecular pathways: the balance between cancer and the immune system challenges the therapeutic specificity of targeting nuclear factor-kappaB signaling for cancer treatment. *Clin Cancer Res.* (2016) 22:4302–8. 10.1158/1078-0432.ccr-15-1374 27422962 PMC5010470

[66] Kim EK Choi EJ. Compromised MAPK signaling in human diseases: an update. *Arch Toxicol.* (2015) 89:867–82. 10.1007/s00204-015-1472-2 25690731

[67] Wei C Cao Y Yang X Zheng Z Guan K Wang Q Elevated expression of TANK-binding kinase 1 enhances tamoxifen resistance in breast cancer. *Proc Natl Acad Sci USA.* (2014) 111:E601–10. 24449872 10.1073/pnas.1316255111 PMC3918824

[68] Yu S Cai X Wu C Liu Y Zhang J Gong X Targeting HSP90-HDAC6 regulating network implicates precision treatment of breast cancer. *Int J Biol Sci.* (2017) 13:505–17. 10.7150/ijbs.18834 28529458 PMC5436570

[69] Buchert M Burns CJ Ernst M. Targeting JAK kinase in solid tumors: emerging opportunities and challenges. *Oncogene.* (2016) 35:939–51. 10.1038/onc.2015.150 25982279

[70] Zhang F Zheng J Kang X Deng M Lu Z Kim J Inhibitory leukocyte immunoglobulin-like receptors in cancer development. *Sci China Life Sci.* (2015) 58:1216–25. 10.1007/s11427-015-4925-1 26566804

[71] Brownlie RJ Garcia C Ravasz M Zehn D Salmond RJ Zamoyska R Resistance to TGFbeta suppression and improved anti-tumor responses in CD8(+) T cells lacking PTPN22. *Nat Commun.* (2017) 8:1343. 10.1038/s41467-017-01427-1 PMC5676842 29116089

[72] Manguso RT Pope HW Zimmer MD Brown FD Yates KB Miller BC In vivo CRISPR screening identifies Ptpn2 as a cancer immunotherapy target. *Nature.* (2017) 547:413–8. 10.1038/nature23270 28723893 PMC5924693

[73] Younis S Javed Q Blumenberg M. Transcriptional changes associated with resistance to inhibitors of epidermal growth factor receptor revealed using metaanalysis. *BMC Cancer.* (2015) 15:369. 10.1186/s12885-015-1337-3 25948104 PMC4430867

[74] Jang JY Kim YG Nam SJ Keam B Kim TM Jeon YK Targeting adenine nucleotide translocase-2 (ANT2) to overcome resistance to epidermal growth factor receptor tyrosine kinase inhibitor in non-small cell lung cancer. *Mol Cancer Ther.* (2016) 15:1387–96. 10.1158/1535-7163.mct-15-0089 26883272

[75] Campia I Buondonno I Castella B Rolando B Kopecka J Gazzano E An autocrine Cytokine/JAK/STAT-signaling induces kynurenine synthesis in multidrug resistant human cancer cells. *PLoS One.* (2015) 10:e0126159. 10.1371/journal.pone.0126159 25955018 PMC4425697

[76] Scagliola A Mainini F Cardaci S. The TCA cycle at the crossroad between cancer and immunity. *Antioxid Redox Signal.* (2020) 32:834–52. 10.1089/ars.2019.7974 31847530

[77] Icard P Shulman S Farhat D Steyaert JM Alifano M Lincet H. How the Warburg effect supports aggressiveness and drug resistance of cancer cells? *Drug Resist Updat.* (2018) 38:1–11. 10.1016/j.drup.2018.03.001 29857814

[78] Ramapriyan R Caetano MS Barsoumian HB Mafra ACP Zambalde EP Menon H Altered cancer metabolism in mechanisms of immunotherapy resistance. *Pharmacol Ther.* (2019) 195:162–71. 10.1016/j.pharmthera.2018.11.004 30439456

[79] Vaupel P Schmidberger H Mayer A. The Warburg effect: essential part of metabolic reprogramming and central contributor to cancer progression. *Int J Radiat Biol.* (2019) 95:912–9. 10.1080/09553002.2019.1589653 30822194

[80] Cascone T McKenzie JA Mbofung RM Punt S Wang Z Xu C Increased tumor glycolysis characterizes immune resistance to adoptive T cell therapy. *Cell Metab.* (2018) 27:977–87.e974. 29628419 10.1016/j.cmet.2018.02.024 PMC5932208

[81] Molino S Tate E McKillop WM Medin JA. Sphingolipid pathway enzymes modulate cell fate and immune responses. *Immunotherapy.* (2017) 9:1185–98. 10.2217/imt-2017-0089 29067886

[82] Kurimoto R Iwasawa S Ebata T Ishiwata T Sekine I Tada Y Drug resistance originating from a TGF-beta/FGF-2-driven epithelial-to-mesenchymal transition and its reversion in human lung adenocarcinoma cell lines harboring an EGFR mutation. *Int J Oncol.* (2016) 48:1825–36. 10.3892/ijo.2016.3419 26984042 PMC4809654

[83] O’Donnell JS Massi D Teng MWL Mandala M. PI3K-AKT-mTOR inhibition in cancer immunotherapy, redux. *Semin Cancer Biol.* (2018) 48:91–103. 10.1016/j.semcancer.2017.04.015 28467889

[84] Lue HW Podolak J Kolahi K Cheng L Rao S Garg D Metabolic reprogramming ensures cancer cell survival despite oncogenic signaling blockade. *Genes Dev.* (2017) 31:2067–84. 10.1101/gad.305292.117 29138276 PMC5733498

[85] Hua H Kong Q Zhang H Wang J Luo T Jiang Y. Targeting mTOR for cancer therapy. *J Hematol Oncol.* (2019) 12:71. 10.1186/s13045-019-0754-1 PMC6612215 31277692

[86] Clark CA Gupta HB Curiel TJ. Tumor cell-intrinsic CD274/PD-L1: a novel metabolic balancing act with clinical potential. *Autophagy.* (2017) 13:987–8. 10.1080/15548627.2017.1280223 28368722 PMC5446070

[87] Ascierto ML McMiller TL Berger AE Danilova L Anders RA Netto GJ The intratumoral balance between metabolic and immunologic gene expression is associated with Anti-PD-1 response in patients with renal cell carcinoma. *Cancer Immunol Res.* (2016) 4:726–33. 10.1158/2326-6066.cir-16-0072 27491898 PMC5584610

[88] Cui W Liu Y Weinstein JS Craft J Kaech SM. An interleukin-21-interleukin-10-STAT3 pathway is critical for functional maturation of memory CD8+ T cells. *Immunity.* (2011) 35:792–805. 10.1016/j.immuni.2011.09.017 22118527 PMC3431922

[89] Gattinoni L Zhong XS Palmer DC Ji Y Hinrichs CS Yu Z Wnt signaling arrests effector T cell differentiation and generates CD8+ memory stem cells. *Nat Med.* (2009) 15:808–13. 10.1038/nm.1982 19525962 PMC2707501

[90] Vander Heiden MG Cantley LC Thompson CB. Understanding the Warburg effect: the metabolic requirements of cell proliferation. *Science.* (2009) 324:1029–33. 10.1126/science.1160809 19460998 PMC2849637

[91] Han F Li G Dai S Huang J. Genome-wide metabolic model to improve understanding of CD4(+) T cell metabolism, immunometabolism and application in drug design. *Mol Biosyst.* (2016) 12:431–43. 10.1039/c5mb00480b 26646474

[92] Angelin A Gil-de-Gómez L Dahiya S Jiao J Guo L Levine MH Foxp3 reprograms T cell metabolism to function in low-glucose, high-lactate environments. *Cell Metab.* (2017) 25:1282–93.e1287. 28416194 10.1016/j.cmet.2016.12.018 PMC5462872

[93] Ohta A. A metabolic immune checkpoint: adenosine in tumor microenvironment. *Front Immunol.* (2016) 7:109. 10.3389/fimmu.2016.00109 27066002 PMC4809887

[94] Xie H Hanai J Ren JG Kats L Burgess K Bhargava P Targeting lactate dehydrogenase–a inhibits tumorigenesis and tumor progression in mouse models of lung cancer and impacts tumor-initiating cells. *Cell Metab.* (2014) 19:795–809. 10.1016/j.cmet.2014.03.003 24726384 PMC4096909

[95] Cerezo M Tomic T Ballotti R Rocchi S. Is it time to test biguanide metformin in the treatment of melanoma? *Pigment Cell Melanoma Res.* (2015) 28:8–20. 10.1111/pcmr.12267 24862830

[96] Zhang Y Kurupati R Liu L Zhou XY Zhang G Hudaihed A Enhancing CD8(+) T cell fatty acid catabolism within a metabolically challenging tumor microenvironment increases the efficacy of melanoma immunotherapy. *Cancer Cell.* (2017) 32:377–91.e379. 28898698 10.1016/j.ccell.2017.08.004 PMC5751418

[97] Carr EL Kelman A Wu GS Gopaul R Senkevitch E Aghvanyan A Glutamine uptake and metabolism are coordinately regulated by ERK/MAPK during T lymphocyte activation. *J Immunol.* (2010) 185:1037–44. 10.4049/jimmunol.0903586 20554958 PMC2897897

[98] Munn DH Sharma MD Baban B Harding HP Zhang Y Ron D GCN2 kinase in T cells mediates proliferative arrest and anergy induction in response to indoleamine 2,3-dioxygenase. *Immunity.* (2005) 22:633–42. 10.1016/j.immuni.2005.03.013 15894280

[99] Araki K Turner AP Shaffer VO Gangappa S Keller SA Bachmann MF mTOR regulates memory CD8 T-cell differentiation. *Nature.* (2009) 460:108–12. 19543266 10.1038/nature08155 PMC2710807

[100] Nakaya M Xiao Y Zhou X Chang JH Chang M Cheng X Inflammatory T cell responses rely on amino acid transporter ASCT2 facilitation of glutamine uptake and mTORC1 kinase activation. *Immunity.* (2014) 40:692–705. 10.1016/j.immuni.2014.04.007 24792914 PMC4074507

[101] Gao P Tchernyshyov I Chang TC Lee YS Kita K Ochi T c-Myc suppression of miR-23a/b enhances mitochondrial glutaminase expression and glutamine metabolism. *Nature.* (2009) 458:762–5. 10.1038/nature07823 19219026 PMC2729443

